# The Construction of Iodine-Doped Carbon Nitride as a Metal-Free Nanozyme for Antibacterial and Water Treatment

**DOI:** 10.3390/nano14161369

**Published:** 2024-08-21

**Authors:** Xinru Cai, Tongtong Xie, Linshan Luo, Xiting Li

**Affiliations:** Hospital of Stomatology, Sun Yat-sen University, Guangzhou 510275, China; caixr3@mail2.sysu.edu.cn (X.C.); xiett@mail2.sysu.edu.cn (T.X.); luolsh7@mail2.sysu.edu.cn (L.L.)

**Keywords:** iodine-doped carbon nitride (I-CN), photocatalysis, antibacterial, Fenton reaction, decontamination

## Abstract

Metal-free photocatalysis that produces reactive oxygen species (ROS) shows significant promising applications for environmental remediation. Herein, we constructed iodine-doped carbon nitride (I-CN) for applications in the photocatalytic inactivation of bacteria and the heterogeneous Fenton reaction. Our findings revealed that I-CN demonstrates superior photocatalytic activity compared to pure CN, due to enhanced light adsorption and a narrowed band gap. Antibacterial tests confirmed that I-CN exhibits exceptional antibacterial activity against both *Escherichia coli* and *Staphylococcus aureus*. The results showed that I-CN effectively generates superoxide radicals and hydroxyl radicals under light irradiation, resulting in enhanced antibacterial activity. In addition, I-CN can also be applied for a heterogeneous photo-Fenton-like reaction, achieving a high performance for the degradation of sulfamethoxazole (SMX), a typical antibiotic, via the photocatalytic activation of peroxymonosulfate (PMS). These results shed new light on the fabrication of metal-free nanozymes and their applications for disinfection and water decontamination.

## 1. Introduction

Dental plaque bacteria that infiltrate periodontal tissues can cause periodontitis, a chronic inflammatory disease [1]. As periodontitis advances, it typically results in the deterioration of the supporting structures of the teeth, the formation of pockets around the teeth, the loosening of teeth, and gum recession, eventually resulting in tooth loss [2]. The conventional clinical approach to treating periodontitis involves a combination of mechanical debridement and antibiotic therapy. Mechanical debridement removes bacteria from periodontal pockets, followed by the use of antibiotics to dissolve the biofilm [3]. Nevertheless, this method often results in gum damage, bleeding, and patient discomfort. Additionally, antibiotics tend to act slowly and can be easily washed away in the oral cavity, necessitating frequent doses [4]. Furthermore, the overuse of antibiotics can contribute to the development of bacterial resistance [5]. Thus, it is essential to implement a safe and effective method for rapid antibacterial treatment.

Photocatalysis is commonly employed for photocatalytic hydrogen and oxygen generation, CO_2_ reduction, and environmental pollution remediation [6,7,8]. Under certain lighting conditions, photocatalysis can generate electron–hole pairs, which participate in redox reactions with surrounding water molecules. This process produces substantial amounts of reactive oxygen species (ROS), including singlet oxygen (^1^O_2_), superoxide radicals (O_2_^•−^), hydroxyl radicals (•OH), and hydrogen peroxide (H_2_O_2_), all of which possess antibacterial properties [9]. The bacterial cells can be swiftly invaded by these ROS, which can then damage their DNA and RNA and ultimately cause the cells to die [10,11]. For example, Lv et al. used an in situ synthesis and hydrothermal approach to generate a new CoS_2_/ZnIn_2_S_4_ (CS/ZIS) photocatalytic antibacterial agent. Within 20 min, the synthesized CS/ZIS was able to inactivate 99.8% of *Escherichia coli* (*E. coli*) caused by ROS [12]. However, the efficiency of single photocatalytic degradation reactions remains a bottleneck. Bi_2_MoO_6_, although capable of absorbing visible light due to its band gap (around 2.5–2.7 eV), suffers from high photocorrosion and the rapid recombination of photogenerated carriers, reducing its effectiveness [13]. To address these issues, combining persulfate advanced oxidation technology (SRAOP) with photocatalytic reactions has been explored for pollutant removal and bacterial inactivation. Somaye Akbari et al. prepared N-doped MgO@Fe_3_O_4_ for Imidacloprid (IMD) degradation and bacterial inactivation via visible light-driven photocatalytic activation of peroxymonosulfate (PMS) [14]. The integration of visible light-driven photocatalysis with heterogeneous catalytic oxidation significantly boosts catalytic activity. Notably, photogenerated electrons (e^−^) and holes (h^+^) can interact with PMS, effectively integrating visible light into the PMS activation process and promoting charge separation. Consequently, advancing visible light-driven photocatalysis for PMS activation offers a promising strategy for improving the degradation of persistent organic pollutants and bacterial inactivation.

Graphitic carbon nitride (CN) is a metal-free semiconductor that is inexpensive, plentiful in the earth, and eco-friendly. However, the limitations of slow charge transfer kinetics and quick charge recombination continue to restrict the effectiveness of metal-free photocatalysts based on CN [15]. To overcome these limitations, non-metallic halogens like F, Cl, Br, and I have been employed to enhance the photoactivity of CN through substitutional or interstitial doping. Iodine can act as an electron donor by transferring electrons to the CN substrate, improving the photocatalytic activity of CN. Furthermore, iodine is present globally in the natural environment and is the most ecologically and physiologically benign element of all of the elements in the halogen family [16]. Iodine-containing antibacterial agents have been widely recognized in the medical and health fields due to their advantages of high efficiency, broad spectrum, and low cost [17]. They are extensively used in infection treatment, medical disinfection, and sewage purification [18]. Additionally, iodine-doped materials have shown potential as nanozymes due to their enzyme-like catalytic properties, enabling efficient ROS generation for antibacterial and pollutant degradation applications [19]. Nanozymes are nanomaterials that mimic the catalytic functions of natural enzymes, exhibiting activities such as oxidase, peroxidase, and superoxide dismutase. These properties allow nanozymes to facilitate reactions that produce ROS, which are crucial for applications in disinfection and environmental remediation [20,21].

Based on the analysis mentioned above, we have selected iodine as the dopant for our study. Herein, we designed and synthesized I-CN by doping iodine into CN. Our findings show that I-CN extends the absorption range and has a stronger photocatalytic capacity compared to the original CN. Antibacterial studies have confirmed that I-CN has excellent antimicrobial activity against both colon and yellow grape globules. This is due to the increased catalytic activity of I-CN when exposed to visible light, which promotes the production of •OH and O_2_^•−^, thereby enhancing the antibacterial effect. Various experiments, such as electron paramagnetic resonance (EPR) spectroscopy, have confirmed the photocatalytic antimicrobial mechanism of I-CN. I-CN functions as a nanozyme, effectively mimicking enzyme-like activities to generate ROS, resulting in superior photocatalytic antimicrobial efficiency. I-CN also demonstrates significant potential as an environmentally friendly marine anti-pollutant and is low in cell toxicity. The use of I-CN in the treatment of periodontitis is an innovative and potentially useful application.

## 2. Experimental Methods

### 2.1. Catalyst Preparation and Characterization

The chemicals used in this study are listed in Text S1, Supporting Information. The polymer precursor was prepared by combining polyethyleneimine (PEI), melamine (M), and cyanuric acid (CA) in an aqueous solution, as outlined in prior work [22]. To synthesize I-CN, an ethanol solution containing 0.5 g of NH_4_I was gradually added to 1.0 g of the polymer precursor (PEI-MCA). The mixture was thoroughly ground, then subjected to a programmed calcination process at a heating rate of 5 °C/min and maintained at 550 °C for 2 h under a nitrogen atmosphere. After cooling to room temperature, the I-CN powder was collected. The synthesis of CN followed the same procedure but used pure PEI-MCA as the starting material. The characterization methods are described in Text S2, Supplementary Materials.

### 2.2. Antibacterial Performance

The antibacterial performance was assessed using the spread plate method. All culture medium (Tryptic Soy Broth (TSB) medium) solutions and apparatus were sterilized. *Escherichia coli* (*E. coli*) and *Staphylococcus aureus* (*S. aureus*) served as model bacterial strains. The overnight cultures of *E. coli* and *S. aureus* were removed from the TSB medium by centrifugation, and then diluted with sterilized saline solution to an optical density (OD) of 0.1 at 625 nm, forming the final bacterial suspension. The bacterial suspensions of *E. coli* and *S. aureus* were mixed with an I-CN aqueous solution (2 mg/mL) to reach a final concentration of 0.55 mg/mL I-CN, and then exposed to simulated visible light irradiation (500 W Xenon lamp) or kept in the dark for 30 min. The bactericidal effect of I-CN was evaluated at various irradiation times (40 min, 80 min, 120 min, 160 min), with 30 min of dark exposure as a control. After irradiation, 100 μL of the illuminated bacterial solution was spread onto solid TSB medium and incubated for 18 h. Each experiment was performed in triplicate. The number of colonies was used to calculate the antibacterial rates using the following equation:

R = (A − B)/A × 100%

where R represents the antibacterial rate, A is the number of colonies in the control group, and B is the number of colonies in the illuminated group.

Furthermore, the Kirby–Bauer method was also used to evaluate the antibacterial activity. A liquid culture medium was used to prepare 0.55 mg/mL of I-CN samples, followed by ultrasonic treatment for 30 min. The blank drug-sensitive paper (6 mm) was soaked in I-CN liquid for about 30 min. The overnight cultures of *E. coli* and *S. aureus* were separated from the TSB medium by centrifugation and then diluted with sterilized saline solution to an optical density (OD) of 0.1 at 625 nm, creating the final bacterial suspension. Next, 100 μL of this bacterial solution was spread onto solid TSB medium and allowed to dry for approximately 15 min. The soaked paper pieces were attached to the surface of the solid culture medium and illuminated for 4 h. The plate was inverted and incubated in an incubator for 18 h. Vernier calipers were used to measure the size of the antibacterial zone, and the experimental results were processed in parallel.

### 2.3. Experimental Procedures

Typically, a 30 mL reaction solution containing 10 mg/L of sulfamethoxazole (SMX) and 0.6 g/L of I-CN was stirred in the dark for 60 min to achieve adsorption/desorption equilibrium. The pH of the solution was adjusted using 0.5 M HCl and 0.5 M NaOH. Then, 0.3 g/L of PMS solution was added to the reaction mixture, which was then exposed to light irradiation from a 300 W xenon lamp (PLS-FX300HU, PerfectLight, Beijing, China) equipped with a UV cutoff filter (λ < 420 nm). At specified time intervals, 500 μL samples of the reaction solution were taken and quenched with 100 μL of methanol. The catalyst was removed by filtration using a 0.22 μm membrane filter. The concentration of SMX was determined using high-performance liquid chromatography (HPLC, LC20AT Shimadzu, Tokyo, Japan), with a mobile phase composed of methanol and water in a 50:50 (*v*/*v*) ratio.

## 3. Results and Discussion

### 3.1. Characterization of I-CN

**Figure 1:**
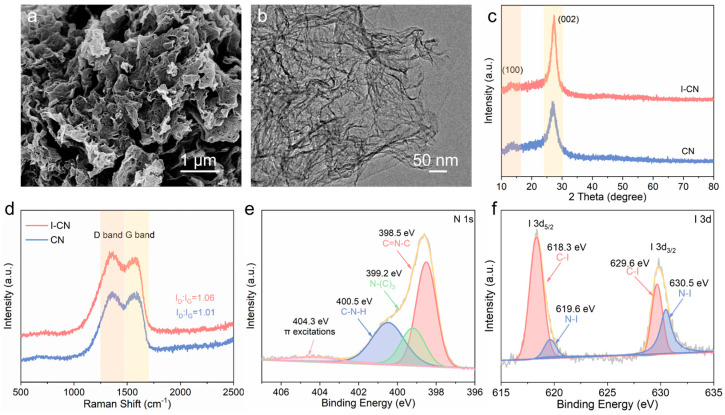
(**a**) SEM image of I-CN; (**b**) TEM image of I-CN; (**c**) XRD patterns; (**d**) Raman spectra of I-CN and CN; (**e**) high-resolution N 1s XPS spectra; (**f**) high-resolution XPS I 3d spectra of I-CN.

The morphology and structure of I-CN were elucidated using scanning electron microscopy (SEM) and transmission electron microscopy (TEM). As illustrated in Figure 1a, the microscopic morphology of the prepared I-CN shows irregularly coiled nanosheets with porous structures, maintaining the basic structure of CN on the whole shown in the previous study [23]. The TEM image of I-CN (Figure 1b) shows similar characterization to CN. It clearly shows that I-CN has a coiled sheet structure with numerous pores and surface defects, which facilitate the mass transfer process and thereby enhance the photocatalytic reaction.

X-ray diffraction (XRD) patterns were employed to analyze the crystal structure of the samples (Figure 1c). The XRD pattern of CN exhibits two distinctive peaks at 13.0° and 27.4°, corresponding to the intralayer repeated packing heptazine units (100) and the interlayer stacking (002) of CN (JCPDS 87-1526), respectively [24]. Therefore, I-CN possesses the exact same framework and crystal structure as pure CN. The Raman spectra of I-CN and CN (Figure 1d) display two wide bands at about 1585 and 1353 cm^−1^, which correspond to the G and D bands, respectively. The I_D_/I_G_ intensity ratio can be utilized to assess the level of graphitization or the density of faulty sites. The I_D_/I_G_ value of 1.06 for I-CN is marginally greater than that of 1.01 for CN, suggesting the creation of an increased number of active sites, thereby facilitating charge accumulation [25].

X-ray photoelectron spectroscopy (XPS) was conducted to elucidate the surface electronic states of the synthesized I-CN. As depicted in Figure S1, the coexistence of C, N, and I elements can be detected in the XPS spectrum of I-CN. The C 1s XPS spectrum (Figure S2) exhibits three peaks at 288.1 eV, 286.4 eV, and 284.9 eV. The peak at 288.1 eV corresponds to the N=C-N bond within the CN framework. The smaller peak at 286.4 eV is attributed to C-NH_x_, while the peak at 284.9 eV is typically associated with the C–C bond in CN [26,27]. Deconvolution of the N 1s spectrum (Figure 1e) reveals four peaks located at 398.5, 399.2, 400.5, and 404.3 eV. These peaks correspond to sp^2^-hybridized nitrogen in C-N=C groups of triazine units, tertiary nitrogen (N-(C)_3_) groups, and free amino groups (C-N-H) in CN, respectively, with the weak peak at 404.3 eV attributed to charging effects [24,28]. Furthermore, the I 3d spectra provide verification of the existence of C–I (618.3 eV) and N–I (619.6 eV) bonds within the I-CN structure, as depicted in Figure 1f. This observation subsequently confirms that the iodine species were successfully incorporated into the synthetic I-CN samples during the preparation process [15,19].

### 3.2. Photocatalytic Property Characterization and Mechanism Analysis

**Figure 2:**
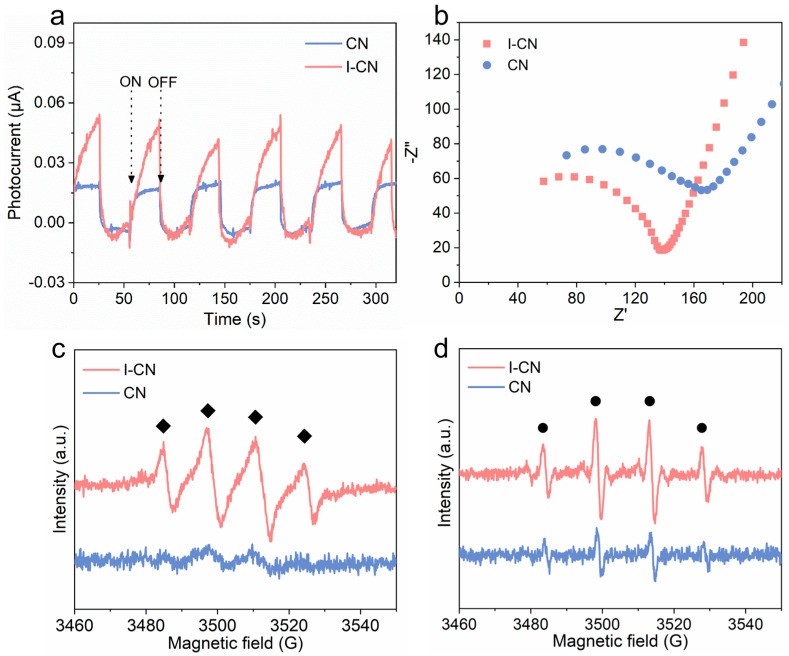
(**a**) Photocurrent response; (**b**) Nyquist diagram of I-CN and CN; DMPO spin-trapping EPR spectra for (**c**) O_2_^•−^ and (**d**) •OH under irradiation-simulated visible light (• represents DMPO–•OH and ◆ represents DMPO–O_2_^•−^).

In general, the transient photocurrent response demonstrates a strong ability to separate photogenerated e^−^ and h^+^ effectively. In accordance with what was anticipated, the photocurrent density of I-CN was significantly greater than that of a single CN (Figure 2a). In addition, the electrochemical impedance spectroscopy (EIS) Nyquist plots presented in Figure 2b illustrate that the arc curve represents the resistance encountered during the electron transfer process on the electrode surface. When comparing CN with I-CN, it is seen that I-CN has a smaller arc radius, indicating a lower interfacial charge transfer resistance. These observations clearly demonstrate that the presence of doped I ions can augment the number of active sites for photocatalysis, facilitate the movement of charge carriers, diminish the pace at which e^−^/h^+^ couples recombine, and eventually improve the efficiency of photocatalytic reactions.

The reactive species were identified using EPR spectroscopy. Figure 2c shows four characteristic peaks for DMPO-O_2_^•−^ in I-CN which were not present in the CN under simulated visible light irradiation, indicating that I-CN has the optimum photocatalytic effect. Similarly, DMPO-•OH signals were detected in I-CN + vis (Figure 2d), further confirming that doped I ions can promote a photocatalytic effect. From the above results, it can be confirmed that I-CN exhibits excellent photocatalytic activity, generating at least two types of ROS under visible light irradiation, which serves as a basis for its sterilization applications. The enzyme-mimicking properties of I-CN as a nanozyme enhance its ability to generate ROS, thus significantly improving its photocatalytic antibacterial performance and environmental remediation capabilities.

**Figure 3:**
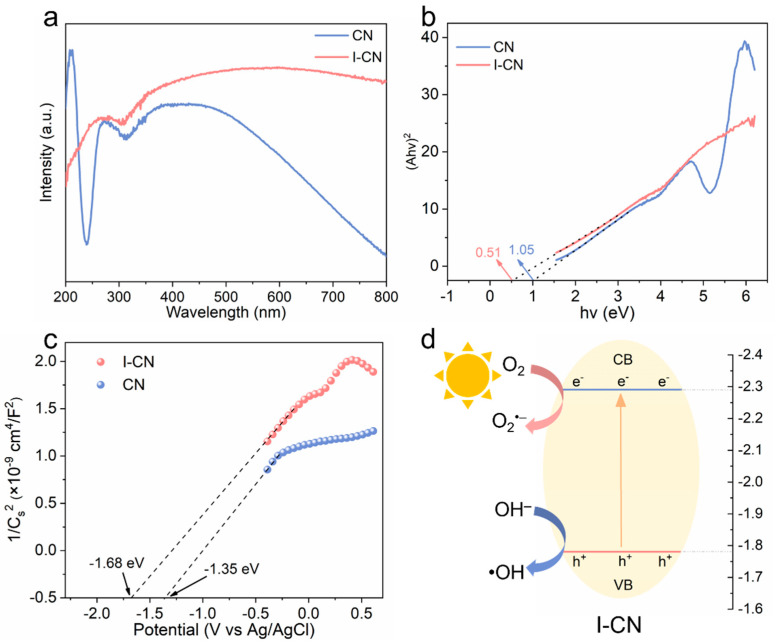
(**a**) UV/Vis DRS spectra, (**b**) Tauc plots, (**c**) Mott–Schottky plots, and (**d**) band structure and photocatalytic mechanism of I-CN.

The UV–Vis–NIR diffuse reflectance spectroscopy spectra of I-CN and CN were analyzed to assess their optical properties and determine the band gap energy (Eg). As illustrated in Figure 3a, the I-CN composite not only expanded the range of wavelengths absorbed, but also significantly increased the overall absorption capacity across the spectrum (200–800 nm) compared to CN alone. This indicates that I-CN has a better ability to absorb visible light than pure CN. Based on the UV–Vis–NIR absorption curve shown in Figure 3b, we determined the Eg of I-CN and CN to be 0.51 and 1.05 eV, respectively. The higher light absorption and reduced band gap of I-CN contribute to the excitation and production of photogenerated carriers, leading to an increase in photocatalytic activity. The Mott–Schottky (M-S) plots for CN and I-CN (Figure 3c) show positive slopes, showing that electrons are the dominant charge carriers (n-type semiconductor) [29]. The conduction band (CB) and valence band (VB) energies of I-CN and CN may be determined simultaneously. The E_VB_ and E_CB_ energies of CN were found to be −1.45 eV and −2.50 eV, respectively. On the contrary, the E_VB_ and E_CB_ energies of I-CN are −1.78 eV and −2.29 eV, respectively [30]. Photocatalysis involves the movement of e^−^ and h^+^. According to the data presented above, we conducted a study on the photocatalytic mechanism. The doping of I ions greatly improves the photocatalytic activity of I-CN. The photocatalytic mechanism of I-CN is illustrated in Figure 3d. The electrons engage in interactions with O_2_ that is adsorbed on the catalyst surface, resulting in the production of O_2_^•−^. This process enhances the efficiency of photocatalysis. Furthermore, the process of directly oxidizing OH^−^ with h^+^ results in the formation of •OH. The results validate that I-CN exhibits excellent photocatalytic capabilities and holds significant potential for application in the treatment of periodontitis.

### 3.3. Antibacterial Ability

**Figure 4:**
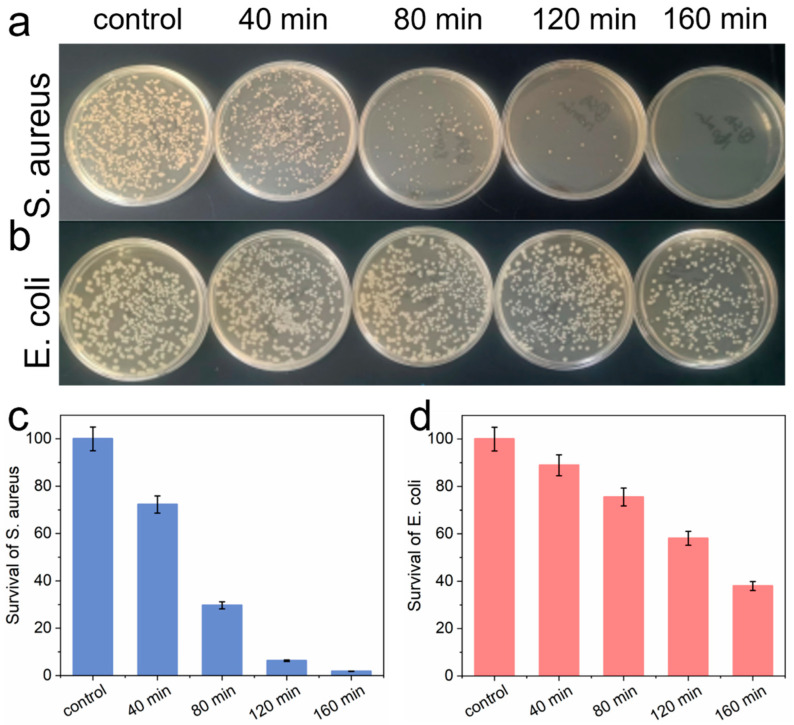
Bacterial colony growth in the presence of I-CN for (**a**) *S. aureus* and (**b**) *E. coli* under different irradiation times; (**c**,**d**) show the survival ratio.

In recent years, several studies have suggested moderate evidence supporting *Escherichia coli* and *Staphylococcus aureus* as periodontal pathogens [31]. Leveraging the superior photocatalytic properties of I-CN, we targeted these pathogenic bacteria and investigated its antibacterial effects on *S. aureus* and *E. coli*. The impact of I-CN on these bacteria was assessed using plate-coating experiments under simulated visible light irradiation at various intervals (Figure 4a,b). As expected, the survival rates of *S. aureus* and *E. coli* decreased progressively with increased illumination time. After 80 min of irradiation, the number of *S. aureus* colonies on the TSB plate was significantly reduced (Figure 4c). When the illumination time reached 160 min, nearly 100% of *S. aureus* was eradicated. These findings suggest that I-CN, functioning as a photocatalyst, undergoes photogenerated electron–hole pair separation upon exposure to visible light. The resulting e^−^ and h^+^ then react with surrounding water molecules and oxygen to produce •OH and O_2_^•−^ radicals. When these free radicals come into contact with bacteria, they rapidly damage the cell wall, leading to the leakage of cell contents and ultimately causing bacterial death [11]. Likewise, the antibacterial efficiency of I-CN against *E. coli* can reach 62.03% after 160 min of irradiation (Figure 4d). The antibacterial efficiency was lower than that of *S. aureus*, but still, there was a significant difference when compared with the control group. To further decipher its antibacterial behavior, the Kirby–Bauer method was also used to evaluate the antibacterial activity. We observed that the diameter of the circle of inhibition of I-CN against *S. aureus* was 0.730, 0.680, 0.700, and 0.764 mm, respectively (Figure S3), and against *E. coli*, it was 0.814, 0.780, 0.806, and 0.884 mm, respectively (Figure S4), all of which, compared to 6 mm in the control group, showed a significant difference, suggesting that I-CN has an obvious antibacterial effect under the effect of light.

Double-staining experiments showed negligible lethality against *S. aureus* and *E. coli* either by light alone or by the addition of I-CN alone (Figures S5 and S6). However, under light conditions, the addition of I-CN increased the mortality of *S. aureus* and *E. coli*. These results suggest that I-CN borrows ROS generated based on its efficient photocatalytic properties to cause bacterial death with the assistance of simulated visible light radiation, indicating the effectiveness of I-CN for periodontitis treatment under visible light irradiation.

**Figure 5:**
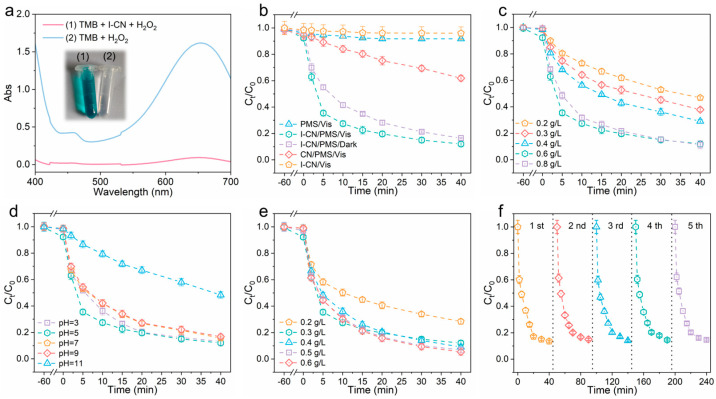
(**a**) Absorption curves of TMB chromogenic reaction catalyzed by I-CN; (**b**) degradation of SMX in various systems; influence of (**c**) catalyst dose, (**d**) PMS dose, and (**e**) initial pH on SMX degradation in I-CN/PMS/Vis system; (**f**) recycling use of I-CN for degradation of SMX via PMS activation over five consecutive cycles. Reaction conditions: [SMX] = 10 mg/L; [catalyst] = 0.6 g/L; [PMS] = 0.3 g/L; initial pH = 5.

Additionally, we evaluated the peroxidase-like activity of I-CN using 3,3′,5,5′-tetramethylbenzidine (TMB) as a probe. The colorimetric assay and UV-Vis absorption spectra (Figure 5a) clearly show that I-CN possesses high peroxidase-like activity, catalyzing the oxidation of colorless TMB into blue-colored TMB oxide in the presence of H_2_O_2_. Consequently, the I-CN nanozyme effectively activates the O-O bond of the peroxide, highlighting its substantial potential in bacterial inactivation applications.

### 3.4. Catalytic Performance of I-CN

We selected SMX as a model emerging organic contaminant to evaluate the catalytic performance of iodine-doped carbon nitride (I-CN) in Fenton-like reactions. The catalytic activity was assessed by examining the degradation of SMX through PMS activation, as depicted in Figure 5b. PMS alone had a negligible oxidation capacity, removing less than 2% of SMX. However, combining the catalyst with PMS significantly reduced the SMX concentration. Notably, the addition of visible light to the I-CN/PMS system greatly enhanced the catalytic performance of I-CN. Optimal dosages for the catalyst and PMS were determined to be 0.6 and 0.3 g/L, respectively (Figure 5c,d). As shown in Figure 5e, the I-CN/PMS/vis system exhibited high SMX degradation efficiencies (>85%) across a wide pH range (3–10). However, under alkaline conditions (pH 11), the removal efficiency of SMX decreased. This reduction is attributed to the negative charge on the surface of SMX, which induces electrostatic repulsion with radicals in an alkaline environment, thereby preventing the interaction between the radicals and SMX. To demonstrate the stability and reusability of the material, we conducted five consecutive cycles of the degradation experiments (Figure 5f). I-CN maintained degradation efficiencies above 82% throughout these cycles, indicating the excellent stability and reusability of the catalyst. In summary, the combination of I-CN with PMS and visible light irradiation significantly enhances the degradation of SMX, demonstrating the potential of this system for treating emerging organic contaminants in diverse environments. In comparison to other materials such as PTh/MnO_2_ [32] and TiO_2_@SiO_2_ [33], our I-CN material offers distinct advantages. PTh/MnO_2_ requires 6 h for significant bacterial inactivation, while our I-CN material achieves rapid antibacterial effects under visible light, making it more practical for clinical applications. Additionally, unlike PTh/MnO_2_ and TiO_2_@SiO_2_, which primarily focus on disinfection, I-CN exhibits high performance in both antibacterial activity and the degradation of residual pharmaceuticals. Furthermore, being a metal-free photocatalyst, I-CN eliminates the risk of metal ion release, ensuring environmental safety and sustainability.

## 4. Conclusions

In this study, we successfully designed and synthesized I-CN by incorporating iodine into CN. I-CN broadens the absorption spectrum, enabling effective photocatalytic sterilization. The iodine doped into the CN substrate promotes the generation of •OH and O_2_^•−^ radicals, thereby enhancing the photocatalytic antibacterial efficiency. EPR spectroscopy confirmed the photocatalytic antibacterial mechanisms of I-CN. Furthermore, we investigated the degradation performance of I-CN on sulfamethoxazole through the synergistic effect of PMS and photocatalysis. The results showed that I-CN alone was insufficient for efficient SMX degradation. However, the introduction of visible light and PMS into the system markedly enhanced the degradation efficiency. The combined effect of photocatalysis and PMS activation greatly enhanced the degradation of SMX, demonstrating the potential of I-CN in environmental remediation applications. Overall, the synthesized I-CN demonstrates remarkable antibacterial activity and substantial promise for various biomedical applications. The nanozyme characteristics of I-CN, which mimic the catalytic functions of natural enzymes, are pivotal in enhancing its performance for ROS generation and pollutant degradation. Its ability to effectively generate ROS and degrade emerging organic contaminants highlights its potential as a versatile and powerful tool in both the environmental and medical fields.

## Data Availability

Data are contained within the article.

## Supplementary Materials

The following supporting information can be downloaded at: https://www.mdpi.com/article/10.3390/nano14161369/s1, Text S1: Chemicals; Text S2: Characterizations; Text S3: Cell double staining; Text S4: Bacterial double staining; Figure S1: XPS survey spectra of I-CN; Figure S2: High resolution C 1s XPS spectra of I-CN; Figure S3: Kirby-Bauer method was used to assess the effect of I-CN on *S. aureus*; Figure S4: Kirby-Bauer method was used to assess the effect of I-CN on *E. coli*; Figure S5: Bacterial double staining method was used to assess the the toxicity of I-CN on *S. aureus* in dark or light conditions. Figure S6: Bacterial double staining method was used to assess the the toxicity of I-CN on *E. coli* in dark or light conditions.
