# Supplementary material for: The Construction of Iodine-Doped Carbon Nitride as a Metal-Free Nanozyme for Antibacterial and Water Treatment
Source: Nanomaterials (Basel). 2024 Aug 21;14(16):1369. doi: 10.3390/nano14161369 (PMC11357014; doi:10.3390/nano14161369)
Supplement: Supplementary file 1 [file nanomaterials-14-01369-s001.zip › nanomaterials-3144337-supplementary.pdf]

Supporting Information

Text S1 Chemicals

Cyanuric Acid ($C_3H_3N_3O_3$) was purchased from Tokyo Chemical Industry Co., Ltd. (Shanghai, China). Melamine ($C_3H_6N_6$), polyethyleneimine (PEI), Methanol (CH_3OH, MeOH), sulfamethoxazole ($C_{10}H_{11}N_3O_3S$, SMX) and Ammonium iodide (NH_4I) were purchased from Aladdin Reagent Co., Ltd. Peroxymonosulfate ($KHSO_5 \cdot 0.5KHSO_4 \cdot 0.5K_2SO_4$) was purchased from Shanghai Macklin Biochemical Co., Ltd. Hydrochloric acid (HCl), sodium hydroxide (NaOH) and sodium sulfate (Na_2SO_4) were purchased from Sinopharm Chemical Reagent Co., Ltd.

Text S2 Characterizations

The morphology of the I-CN was observed by scanning electron microscopy (SEM, Hitachi S-4800, Japan) and transmission electron microscopy (TEM, JEOL-2100F, Japan). The phase of the samples was inspected by X-ray diffraction (XRD, Shimadzu XRD-6000, Japan). The surface elements were analyzed by X-ray photoelectron spectroscopy X-ray photoelectron spectroscopy (XPS, ESCALAB 250, Thermo Fisher Scientific, USA). Raman spectra were collected by using a Horiba Jobin Yvon LabRam instrument with a HeNe laser excitation at 532 nm. Electron spin resonance (EPR) analysis was performed on a Bruker EPR I200 spectrometer.

A series of photoelectrochemical experiments, including the photocurrent responses, electrochemical impedance spectroscopy (EIS), open circuit potential (OCP) and Mott–Schottky plots (M-S plots) of samples were measured by CHI-660E electrochemical workstation (Shanghai Chenhua Co. Ltd., China) with platinum as the counter electrode, Ag/AgCl as the reference electrode, and Na_2SO_4 (0.5 M) as the electrolyte solution. The light source was full spectrum (CEL-HXF300-T3, China). The concentration of the SMX was determined by high performance liquid chromatography (HPLC, LC20AT Shimazu, Japan).

Text S3 Cell double staining

The Calcein-AM/Propidium iodide (Calcein-AM/PI) Double staining Kit (Saint-Bio, China) was utilized for the determination of cell death. The 231 cells were cultured with

or without I-CN (250 μg/mL) for 24 hours under normal or light conditions (660 ± 15 nm). The cultured cells (1 × 10^6 CFU/mL) were stained with a staining solution containing 5 μL Calcein-AM solution, 15 μL PI solution and 5 mL phosphate-buffered saline (PBS). The live cells (chartreuse fluorescence) and dead cells (red fluorescence) were detected with 490 ± 10 nm excitation filter under fluorescence microscope.

Test S4 Bacterial double staining

Under ultrasound conditions (30 minutes), I-CN samples with a concentration of 2.5 mg/mL were prepared using physiological saline. The cultured bacteria were collected and adjusted to an OD of 0.2 using physiological saline. 400 microliters of I-CN sample, 400 μL of bacterial solution and 200 μL physiological saline were mixed evenly and then processed in darkness or light (500W xenon lamp, λ> 420nm) for 160min. The above bacteria were stained in the dark for 15 minutes using a live/dead bacterial double staining kit. In order to fix the bacteria, an agarose solution with a concentration of 10mg/mL was applied to the slide and naturally solidified. A sample of 50 microliters is coated on the surface of solidified agarose. The glass slide is placed under a microscope and observed after being covered with a cover glass.

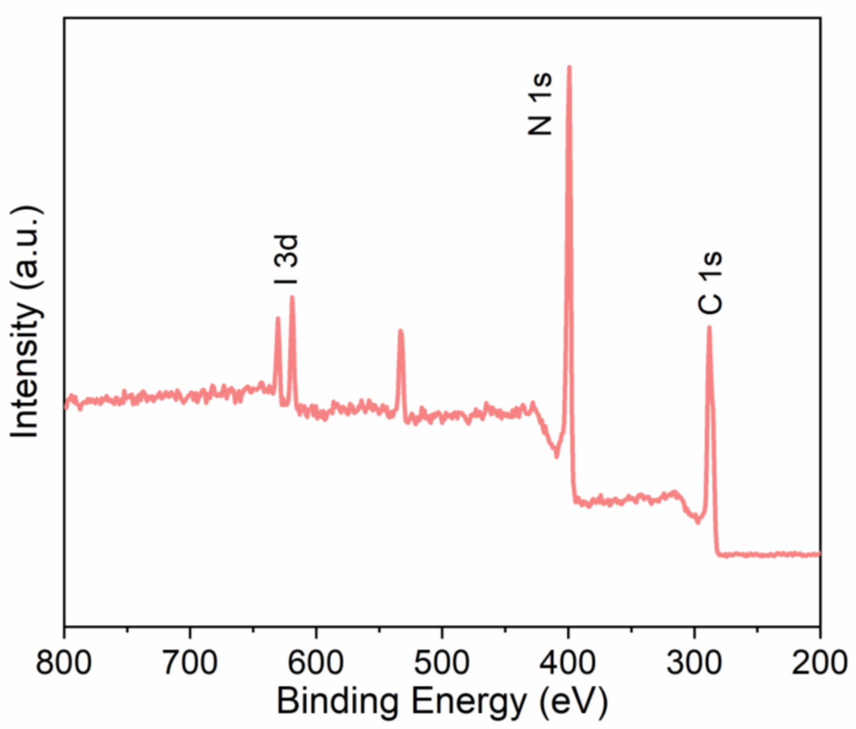

Figure S1. XPS survey spectra of I-CN

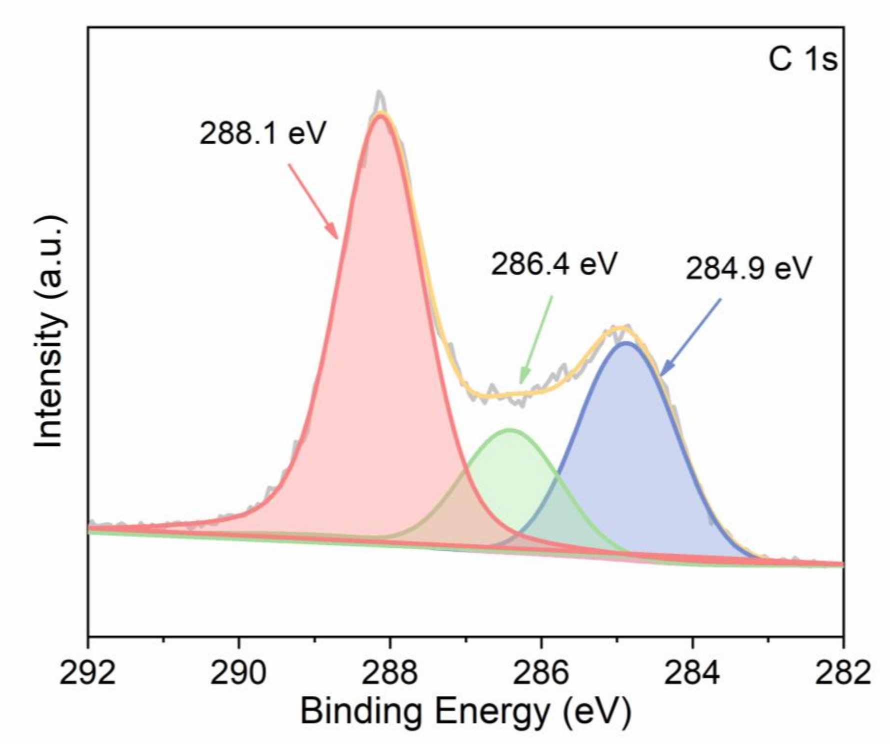

Figure S2. High resolution C 1s XPS spectra of I-CN

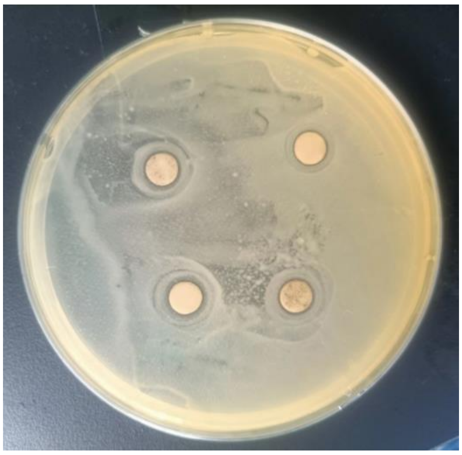

Figure S3. Kirby-Bauer method was used to assess the effect of I-CN on S. aureus

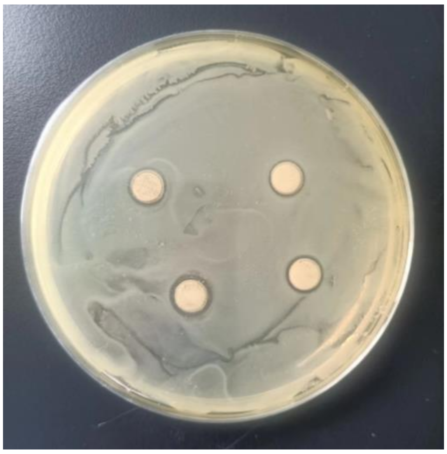

Figure S4. Kirby-Bauer method was used to assess the effect of I-CN on E. coli

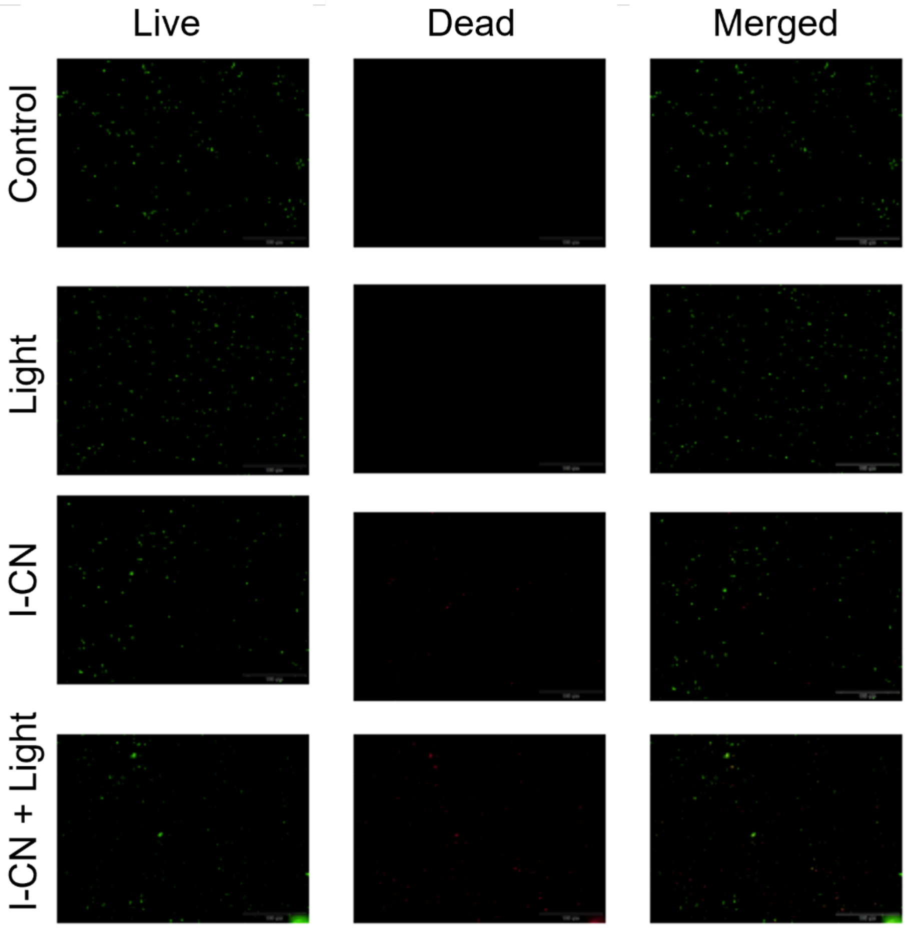

Figure S5. Bacterial double staining method was used to assess the the toxicity of I-CN on S. aureus in dark or light conditions.

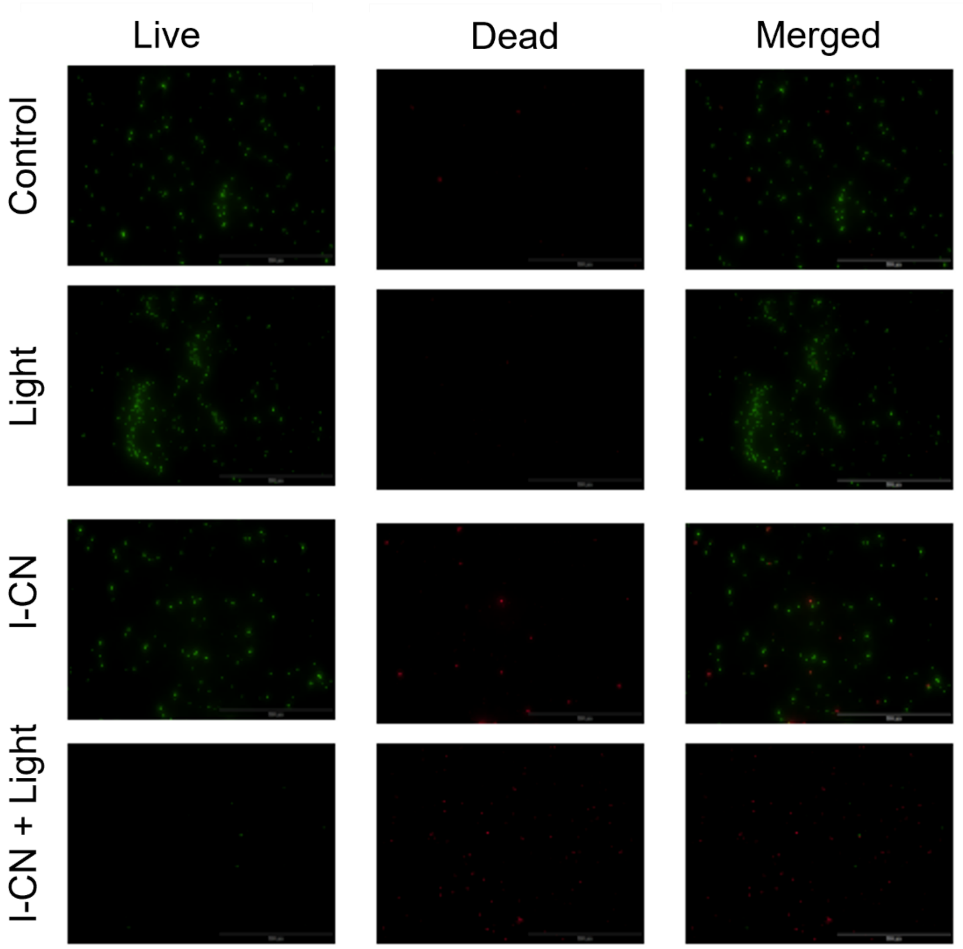

Figure S6. Bacterial double staining method was used to assess the the toxicity of I-CN on E. coli in dark or light conditions.
